# The structure of Human Microplasmin in Complex with Textilinin-1, an Aprotinin-like Inhibitor from the Australian Brown Snake

**DOI:** 10.1371/journal.pone.0054104

**Published:** 2013-01-15

**Authors:** Emma-Karin I. Millers, Lambro A. Johnson, Geoff W. Birrell, Paul P. Masci, Martin F. Lavin, John de Jersey, Luke W. Guddat

**Affiliations:** 1 School of Chemistry and Molecular Biosciences, The University of Queensland, Brisbane, Queensland, Australia; 2 Department of Medicine, Princess Alexandra Hospital, Woolloongabba, Queensland, Australia; 3 The Queensland Cancer Fund Research Unit, The Queensland Institute of Medical Research, Royal Brisbane Hospital, Herston, Brisbane, Queensland, Australia; George Washington University, United States of America

## Abstract

Textilinin-1 is a Kunitz-type serine protease inhibitor from Australian brown snake venom. Its ability to potently and specifically inhibit human plasmin (K_i_ = 0.44 nM) makes it a potential therapeutic drug as a systemic anti-bleeding agent. The crystal structures of the human microplasmin-textilinin-1 and the trypsin-textilinin-1 complexes have been determined to 2.78 Å and 1.64 Å resolution respectively, and show that textilinin-1 binds to trypsin in a canonical mode but to microplasmin in an atypical mode with the catalytic histidine of microplasmin rotated out of the active site. The space vacated by the histidine side-chain in this complex is partially occupied by a water molecule. In the structure of microplasminogen the χ_1_ dihedral angle of the side-chain of the catalytic histidine is rotated by 67° from its “active” position in the catalytic triad, as exemplified by its location when microplasmin is bound to streptokinase. However, when textilinin-1 binds to microplasmin the χ_1_ dihedral angle of this amino acid residue changes by −157° (*i.e.* in the opposite rotation direction compared to microplasminogen). The unusual mode of interaction between textilinin-1 and plasmin explains textilinin-1′s selectivity for human plasmin over plasma kallikrein. This difference can be exploited in future drug design efforts.

## Introduction

Aprotinin (Trasylol®) is a Kunitz-type serine protease inhibitor that has been in broad use for ∼40 years as a therapeutic agent to decrease blood loss in patients undergoing surgical procedures. However, an extensive study conducted by Fergusson and colleagues involving over 2000 high risk cardiac surgery patients showed that its use is associated with a significantly increased risk of stroke, heart failure, myocardial infarction, encephalopathy, and vascular, cardiovascular and cerebrovascular events compared with the lysine analogue anti-bleeding agents, tranexamic acid and ε-amino caproic acid [1]. The study also showed that patients receiving aprotinin as compared to the other treatments were less likely, by ∼3%, to suffer a massive bleeding episode. As a result of the higher risks of side-effects associated with aprotinin its use as an anti-bleeding agent has now been suspended in many countries [2], [3]. Overall, the Fergusson study highlights the need for the discovery of improved anti-bleeding agents that are both safe and highly effective.

Snake venoms are a good source for the discovery of novel therapeutic agents [4], [5]. Kunitz-type inhibitors (similar in structure to aprotinin) are one class of small protein commonly found in such venoms [6]. These molecules can have exquisite binding specificities and possess high potency for their targets making them excellent therapeutic candidates. Textilinin-1, isolated from the venom of the Australian brown snake, *Pseudonaja textilis*, is an experimental drug (Q8008, Venomics Pty Ltd) and is suggested as a novel anti-bleeding agent to replace aprotinin. Possible modes of action for aprotinin include inhibition of plasmin (EC 3.4.21.7) thereby reducing fibrinolysis and/or inhibition of plasma kallikrein (EC 3.4.21.34) limiting activation of inflammatory pathways. Some of aprotinin’s detrimental side-effects [1], [2], [7], [8] may be due to (i) its overall lack of protease specificity, and (ii) its virtually irreversible inhibition of plasmin, leading to prolonged inhibition of fibrinolysis. Textilinin-1 is a good inhibitor of plasmin but with a more narrow binding specificity compared to aprotinin [9], [10]. No measurable inhibition could be detected for textilinin-1 against tissue plasminogen activator, urokinase, activated protein C and elastase while aprotinin inhibits these enzymes with K_i_ values in the micromolar range [9], [10]. Previous studies have shown that the K_i_ ratios in favour of aprotinin over textilinin-1 are 295, 9.9×10^3^ and 1.3×10^4^ for plasma kallikrein, tissue kallikrein and trypsin, respectively [9], [10]. These data suggest that its application as an anti-bleeding agent may not be accompanied by the observed side-effects associated with aprotinin.

An understanding of the structural basis for the inhibition of serine proteases by textilinin-1 is important in developing it or related inhibitors into pharmaceuticals. To date, no structural data have been obtained for a Kunitz/BPTI-type inhibitor in complex with either plasmin or plasma kallikrein. These structures are needed to understand the molecular interactions that are responsible for potency and specificity. Crystallization of complete plasmin with its kringle domains or plasma kallikrein with its apple domains have historically been problematic due to the modular nature and inherent flexibility of these molecules, though recently the first crystal structure of complete plasmin has now become available [11]. However, in both cases it has been possible to determine structures of the catalytic domains alone [12], [13]. Furthermore, crystallization of aprotinin in complex with these protease domains has, to date, proven intractable.

A key structural feature of aprotinin and related Kunitz/BPTI inhibitors is a loop located between the first α-helix and β-sheet of the polypeptide. This region is known as the canonical loop and binds in a complementary manner to the active site of a serine protease but is not cleaved by the enzyme. There are six amino acids in aprotinin that are crucial in binding to serine proteases: P13, C14, K15, A16, R17 and I18. A nomenclature to describe the location of these amino acids has been established identifying these residues generally as P_3_, P_2_, P_1_, P_1_′, P_2_′, P_3_′, respectively [14]. The opposing sites on the protease are signified by the letter S accompanied by the appropriate superscript and subscript. Proteolytic cleavage ordinarily occurs at the peptide bond between the P_1_ and P_1_′ residues. However, this does not happen when a Kunitz/BPTI inhibitor forms a complex with a serine protease. Instead BPTI becomes tightly bound to the enzyme, acting as an inhibitor. The critical amino acids in the active site of the protease are the catalytic triad of a histidine, aspartic acid and a serine (S741 in plasmin and S195 in trypsin) whose side-chain hydroxyl is the nucleophile in the reaction.

Herein, we describe a kinetic study of the inhibition of human plasmin and plasma kallikrein by textilinin-1 and aprotinin, and report the crystal structure of the complex between urokinase-activated microplasmin (protease domain of human plasmin) and textilinin-1. In addition we have determined the crystal structure of the complex between textilinin-1 and bovine trypsin. This structure allows for a discussion of the comparison of the mode of binding of textilinin-1 to two different serine proteases.

## Results

### Inhibition of Plasmin and Plasma Kallikrein by Textilinin-1 and Aprotinin

**Figure 1:**
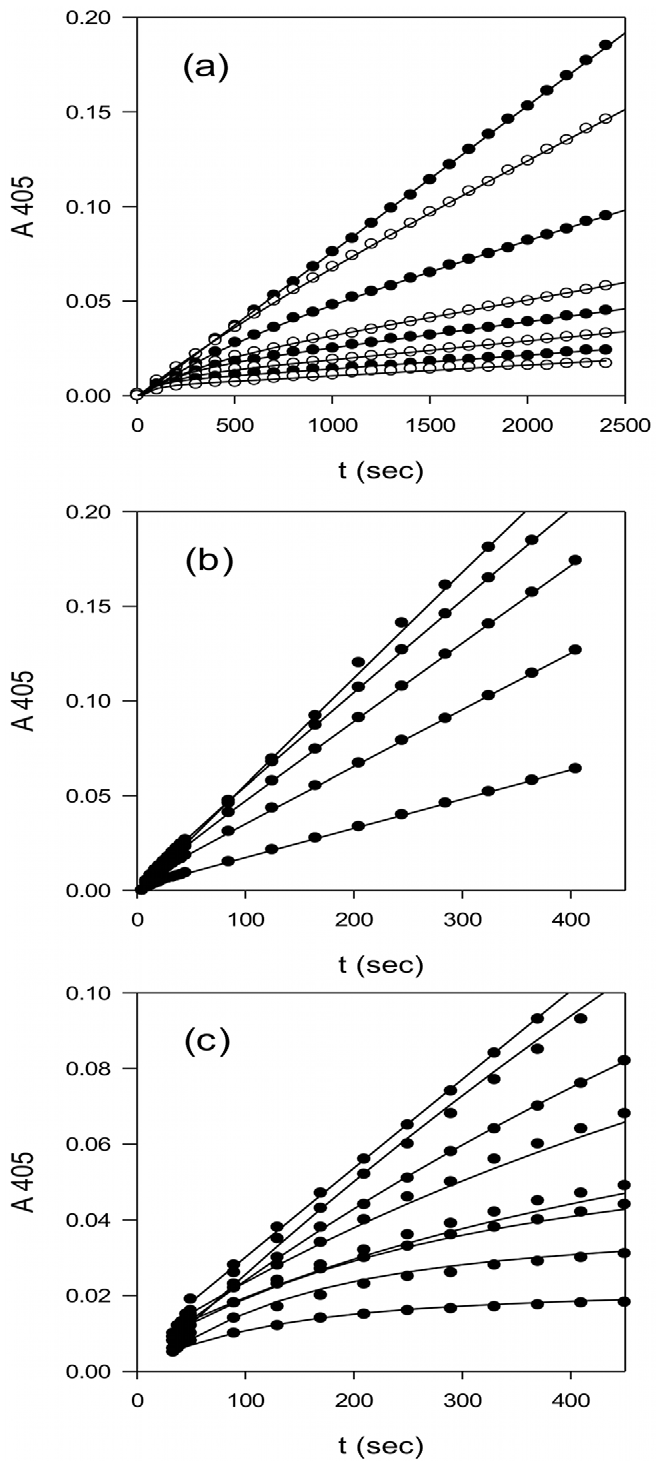
Progress curves for *p*-nitroaniline production by plasmin, plasma kallikrein and trypsin in the presence of textilinin-1. (a) 0.5 nM plasmin in the presence of 0, 2, 5, 10, 15, 20, 40 and 50 nM textilinin-1; (b) 1.0 nM plasma kallikrein in the presence of 0, 3, 6, 12 and 24 nM textilinin-1; and (c) 0.5 nM trypsin in the presence of 0, 2, 3, 4, 6, 11, 19 and 21 nM textilinin-1. For clarity of presentation only a selection of the data points are shown. The fitted lines were obtained by simultaneous regression analysis of each set of curves, using the numerical integration software DynaFit and the one-step model in Scheme 1. The correlation coefficients in each set were >0.99. The resulting parameter estimates (*k_on_* and *k_off_*) and their associated error estimates are shown in Table 1.

Here, we have shown that while textilinin-1 and aprotinin are both potent inhibitors, aprotinin binds about 44, 98 and 698 times more tightly than textilinin-1 to plasmin (K_i_ = 0.01 *vs* 0.44 nM), kallikrein (K_i_ = 19 *vs* 1870 nM) and trypsin (K_i_ = 6×10^−5^ *vs* 0.42 nM) (**Table 1** **;** **Figure 1**). The *k_on_* and t_1/2 (on)_ values for the two inhibitors indicate rapid rates of inhibition. However, the *k_off_* and t_1/2 (off)_ values show that on removal of unbound inhibitor from the system, the activity of textilinin-1 treated plasmin would recover 32 times faster than the activity of aprotinin-treated plasmin. In contrast to aprotinin, textilinin-1 binds relatively weakly to plasma kallikrein with a K_i_ of 1.9 µM (**Table 1**), a slower association (t_1/2 (on)_ = 55 s) and much faster dissociation (t_1/2 (off)_ = 0.49 min). The inhibition parameters in **Table 1** suggest that a therapeutic dose of textilinin-1, which extensively inhibits plasmin (and hence fibrinolysis) while not significantly inhibiting plasma kallikrein should be achievable.

**Table 1:** Kinetic constants for the inhibition of plasmin, kallikrein and trypsin by textilinin-1 and aprotinin.

		textilinin-1				aprotinin			
		plasmin	kallikrein	trypsin		plasmin		kallikrein	Trypsin
*K_i_*	nM	0.436±0.009	1870±630	0.419±0.060		0.010±0.001		19±1.7	6×10^−5^ *
*k_on_*	s^−1^ M^−1^	1.72±0.02×10^6^	1.26±0.43×10^4^	8.60±0.44×10^5^		2.45±0.04×10^6^		3.03±0.17×10^4^	4.13±0.06×10^2^
*k_off_*	s^−1^	7.50±0.11×10^−4^	2.35±0.18×10^−2^	3.60±0.51×10^−4^		2.39±0.25×10^−5^		5.76±0.60×10^−4^	–¶
*t_1/2__(on)_*	s	0.40	55	0.81	0.28		23		1680
*t_1/2__(off)_*	min	15	0.49	32	483		20		–¶

* Taken from Fritz and Wunderer [17].

¶ apparently irreversible inhibition was observed.

*t_1/2__(on)_* is calculated from *k_on_* for an inhibitor concentration of 1 µM.

### Crystal Structure of the Microplasmin-textilinin-1 Complex

The crystal structure of the complex between textilinin-1 and human microplasmin has been determined to 2.78 Å resolution (**Table 2**). Two complexes are present in the asymmetric unit. The overall folds of the protease domain and textilinin-1 are similar to those observed in structures of the separate molecules [12], [15] with the root-mean-squared deviation (rmsd) values upon superimposition of all Cα atoms is ≤0.9 Å. For textilinin-1 in the microplasmin complex, rmsd values for all Cα atoms are in the range 0.40 Å to 0.74 Å when these structures are compared with the three molecules in the asymmetric unit for the free inhibitor (PDB code 3BYB). In general, the major differences in structure occur at the N- and C- termini for the three polypeptides. However, for molecule “C” in the free inhibitor structure the canonical loop adopts a different conformation compared to all of the other textilinin-1 structures. A discussion of this difference in structure is described later in the text. For microplasmin in the textilinin-1 complex, rmsd values for all Cα atoms are in the range 0.55 Å to 0.90 Å when these structures are superimposed on microplasmin in the streptokinase (PDB code 1L4D) and staphylokinase (PDB code 1BUI) complexes. The major differences in structure occur in the loop regions 688–695 and 583–585 and at the N-termini of the polypeptides. None of these regions are directly involved in the interaction between microplasmin and textilinin-1. However, the 688–695 is involved in interactions stabilizing both the streptokinase and staphylokinase complexes, thereby altering its conformation compared to how it exists in the textilinin-1 complex.

**Table 2:** Data collection and refinement statistics for the textilinin-1 complexes.

	textilinin-1	Trypsin
*Diffraction data*		
Temperature (K)	100	100
Resolution Range (Å)	41.60-2.78	37.42-1.64
Observations (I>σ(I))	53,569	192,531
Unique reflections (I>σ(I))	15,858	49,941
Completeness (%)	99.8 (99.3)^a^	97.1 (90.7)
<I/σ(I)>	7.9 (3.6)	22.2 (11.4)
R_sym_^b^	0.117 (0.284)	0.055 (0.494)
*Crystal parameters*		
Solvent content (%)	48	59
Matthews coefficient (Å^3^ Da^-1^)	2.35	3.05
Space group	P2_1_	P3_1_21
Unit cell lengths (Å)	a = 81.40 b = 48.52c = 82.63	a = 79.84 b = 79.84c = 107.39
Unit cell angles (°)	α = 90.0 β = 102.1γ = 90.0	α = 90.0 β = 90.0γ = 120.0
Mosaicity (°)	0.75	0.43
*Polypeptide and other components observed*		
Textilinin-1 in complex A	3–58	3–58
Protease in complex A	547–559 and 562–791	16–245
Textilinin-1 complex B	3–59	No subunit B
Protease in complex B	545–559 and 562–791	No subunit B
H_2_O	107	279
SO_4_^2−^	2	0
Ca^2+^	0	1
*Average B-factors* (Å^2^)		
Textilinin-1 in complex A	25.5	31.7
Protease in complex A	31.4	23.2
Textilinin-1 in complex B	24.3	No subunit B
Protease in complex B	23.2	No subunit B
H_2_O	19.8	35.0
*Refinement*		
R_work_^c^	0.208	0.189
R_free_^c^	0.258	0.215
rmsd bond lengths (Å)	0.004	0.019
rmsd bond angles (^o^)	0.90	1.98
*Ramachandran plot (%)*		
Favoured	94.5	97.5
Outliers	0.5	0.0

a Values in parentheses are for the outer resolution shells 2.89–2.78 Å, and 1.69–1.64 Å for the microplasmin and trypsin complexes, respectively. ^b^ *R*_sym_ = Σ_h_ Σ*_i_* | *I*_h,*i*_ - <*I*_h_>|/Σ_h_ Σ*_i_ I*_ h,*i*_ where *I*_h,i_ is the intensity of the *i*th measurement of reflection h and <*I*_h_> is the average value over multiple measurements. ^c^ *R*_work_ = Σ||F_obs_|-|F_calc_||/Σ|F_obs_|, *R_work_* is calculated based on the reflections used in the refinement (95% of the total data) and *R_free_* is calculated using the remaining 5% of the data.

**Figure 2:**
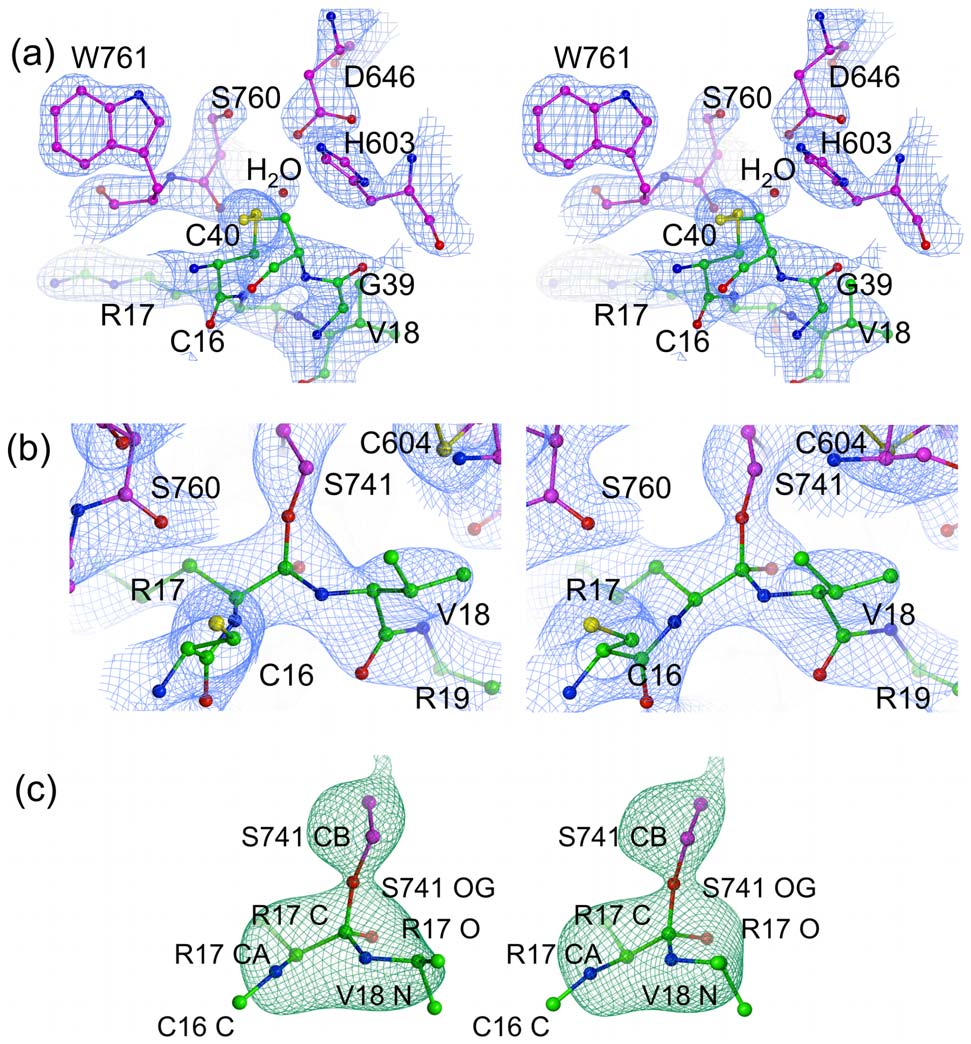
Electron density maps for the microplasmin-textilinin-1 complex. (a) Stereo representation of the 2F_o_-F_c_ electron density (contoured at 1.3 σ) for the interface between textilinin-1 and microplasmin. (b) Stereo representation of the 2F_o_-F_c_ electron density (contoured at 1.3 σ) for the region around R17(textilinin-1) and S741 (microplasmin). (c) omit F_o_-F_c_ electron density map after simulated annealing contoured at 3σ for R17(textilinin-1) and S741 (microplasmin). Textilinin-1 and microplasmin are identified by purple and green carbon atoms, respectively. Bond angles centered around the carbonyl carbon for the tetrahedral intermediates refined to values in the range 103.0° to 118.3° with an average value of 109.3° for both complexes in the asymmetric unit.

**Figure 3:**
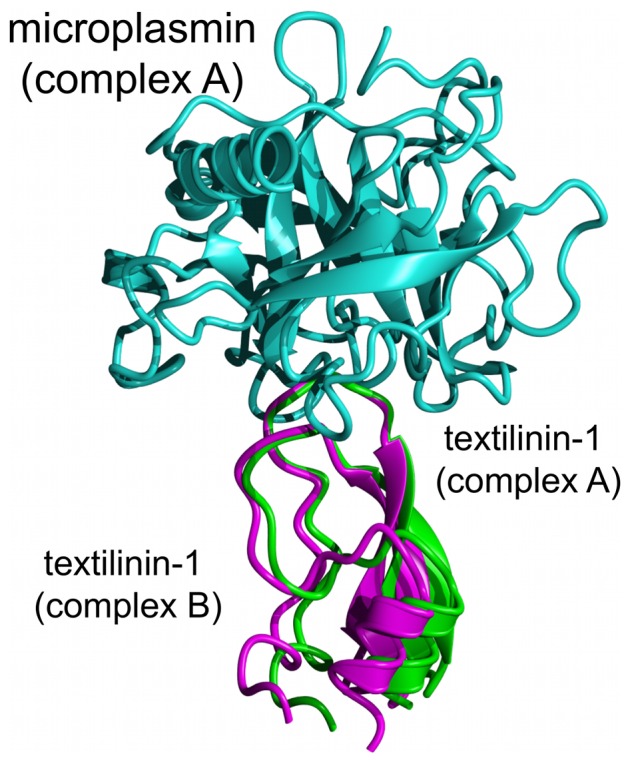
Superimposition of the two complexes in the asymmetric unit of the microplasmin-textilinin-1 crystal structure. The textilinin-1 molecules differ by 8° in their docking angles with microplasmin.

For microplasmin no electron density connects R561 and V562 confirming that the peptide bond between these two residues was, as expected, cleaved by urokinase. The α-amino group of V562 is in the “activated” position making an ionic bond with the side-chain of D740. The electron density maps for the interface between microplasmin and textilinin-1 and for the catalytic centre are presented in **Figure 2**. Textilinin-1 docks into the active site of microplasmin at angles that differ by ∼8° in the two structures in the asymmetric unit (**Figure 3**). This ability to have flexible binding modes suggests that textilinin-1 is not as rigidly locked in place at the active site as occurs in the trypsin-aprotinin [16] complex where the K_i_^*^ value of 0.06 pM is ∼10^4^ lower [17].

**Figure 4:**
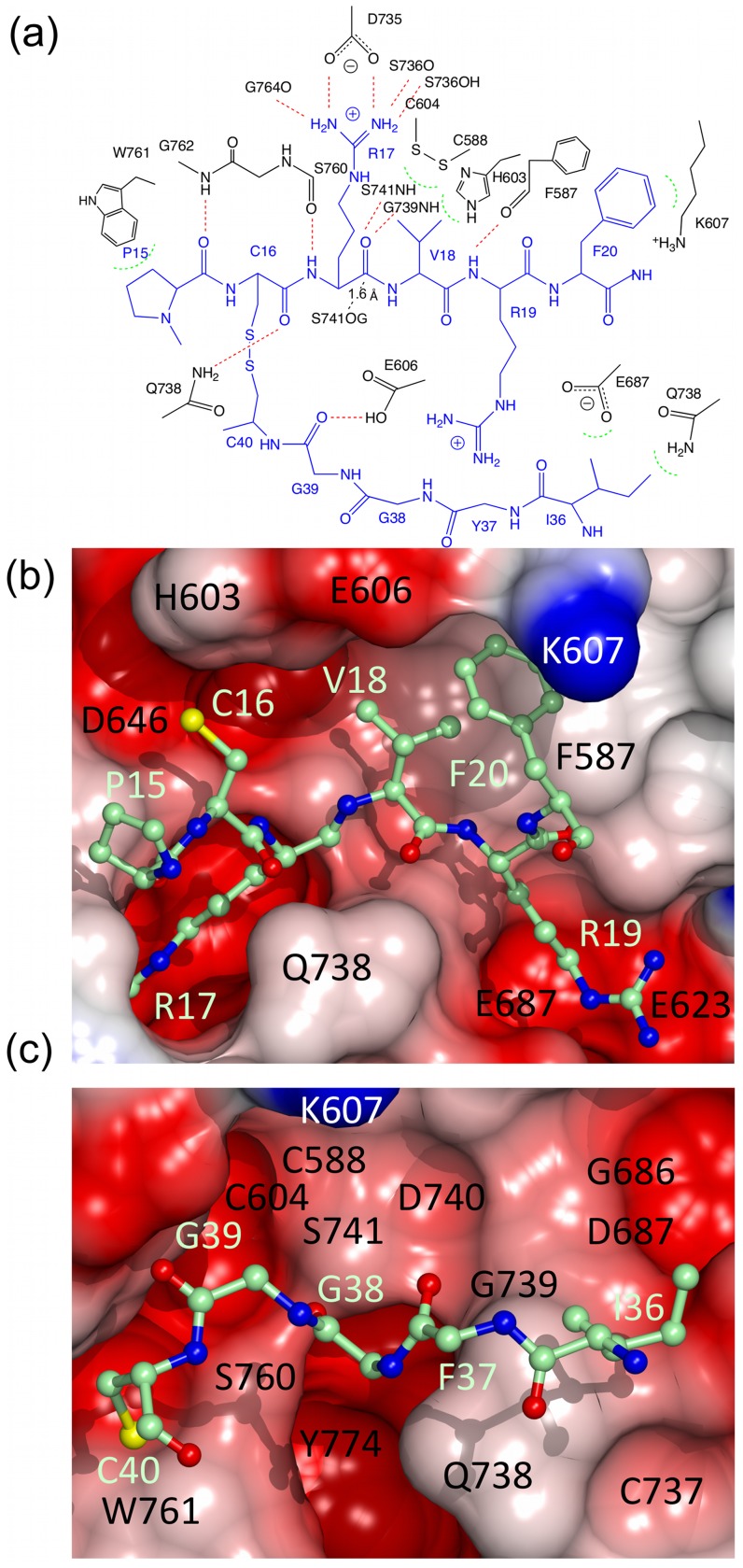
Interactions between microplasmin and textilinin-1. (a) ChemDraw representation of the interactions between microplasmin (black) and textilinin-1 (blue). Hydrogen bonds are shown as red dashed lines. Dashed green semi-circles represent van der Waals attractions. A closer than van der Waals approach of the -OH nucleophile of S741 towards the carbonyl carbon of R17 is depicted as a black dashed line. (b and c) Connolly and electrostatic surface of microplasmin and interactions with textilinin-1 (as observed in complex A in the asymmetric unit of the crystal structure). The canonical loop, including residues P15 to F20 of textilinin-1, and the secondary loop, including residues I36 to C40 are drawn as a stick models. In (c) the side-chain of F37 is removed for clarity.

**Figure 5:**
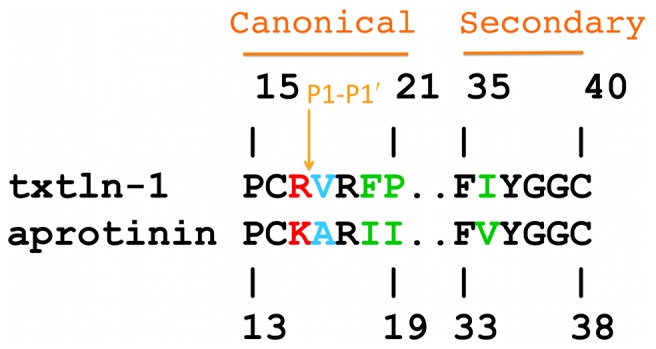
Sequence alignment for the canonical and secondary loops of textilinin-1 and aprotinin. The residue colored red is the P1 site and cyan the P1' site.

**Figure 6:**
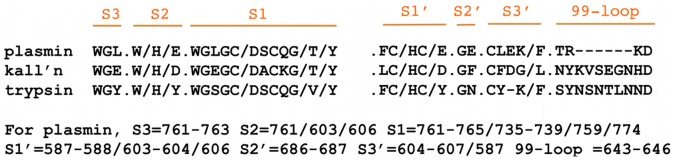
Sequence alignment for the subsites for plasmin and plasma kallikrein and the 99-loop. The 99-loop is a section of polypeptide located behind the catalytic histidine in plasma kallikrein. Similar loops are present in tissue kallikrein, also referred to as KLK1 [43], and trypsin. In plasmin, this loop is missing and none of the near by amino acid residues occupy this space. A “/” represents a break in the polypeptide sequence.

The interactions between microplasmin and textilinin-1 are shown in **Figure 4**. The P3-P3' residues (P15-F20; **Figure 5**) in the canonical loop and I36 to C40 in the secondary binding loop of textilinin-1 make contact with the S3-S3' sites (**Figure 6**) in microplasmin. The overall surface area buried upon formation of the complex is 1360 Å^2^ (*cf.* 1440 Å^2^ for aprotinin and trypsin [16]). As expected, the side-chain from R17 of textilinin-1 (P1 residue) fits into the S1 pocket and forms a salt bridge with D735 of microplasmin. Hydrogen bonds are also formed between the guanidino group of R17 and G764O and S736O and the hydroxyl group of the side-chain of S736. The R17O atom is positioned in the oxyanion hole, and forms hydrogen bonds to G739N and S741N. There is continuous electron density between the catalytic S741OG of microplasmin and R17C of textilinin-1 in both 2F_o_-F_c_ and in simulated annealing omit F_o_-F_c_ maps (**Figures 2b and 2c**). These two atoms are separated by a sub van der Waals distance (1.6 Å) with the carbonyl carbon atom having approximate tetrahedral geometry. The presence of the complex trapped in this tetrahedral intermediate state is consistent with the observation of slow tight binding kinetics (**Figure 1** **;** **Table 1**).

When aprotinin and trypsin make a complex, a short antiparallel β-sheet is formed at the interface between the two partners [18]. The same occurs in the microplasmin-textilinin-1 complex with the residues involved being G762-W761-S760 of microplasmin and the P3-P1 residues, P15-C16-R17, of textilinin-1 (**Figures 4a and 4b**). The hydrogen bonds that stabilize this β-sheet are between P15O of textilinin-1 and G762N of microplasmin and R17N of textilinin-1 and S760O of microplasmin. P15 makes a kink in the structure of textilinin-1, which positions its amide nitrogen too far from the microplasmin surface to form a hydrogen bond. However, this proline is stabilized by hydrophobic contacts to W761 of microplasmin. A hydrogen bond is also formed between C16O of textilinin-1 and the side-chain amide nitrogen of Q738.

The S1' site of microplasmin is partly open and is defined by the side-chain of F587 and the C588-C604 bridge (**Figure 4**). The side-chain of V18 (P1') fits into this site but it has different conformations in the two complexes of the asymmetric unit. This difference in conformation may be the reason for the difference in docking angles observed in the two complexes, though we cannot rule out that the difference in docking angle is not also due to crystal packing effects (see below). The presence of the alternative conformations does however demonstrate that there is flexibility at the interface between microplasmin and textilinin-1. Furthermore, there is not complete complementarity at this site signifying that there could be opportunities to modify the structure to improve affinity and or selectivity. The S2' subsite is partially exposed to solvent with the side-chain of R19 of textilinin-1 in an extended conformation poised over but not firmly packed against any side-chains of microplasmin. Nonetheless, the guanidino group of R19 is loosely held in place by electrostatic attraction to three backbone oxygen atoms and to the side-chains of E623 and E687 (**Figure 4**), which are all within 5 Å but outside hydrogen bonding distance (*i.e.* >3.2 Å). The backbone of R19 is stabilized by a hydrogen bond between its amide nitrogen and F587O of plasmin. The S3' subsite consists mainly of the side-chain of F587 and the hydrophobic part of the side-chain of K607 with one-edge of the side-chain of F20 from textilinin-1 slotting neatly into this pocket (**Figure 4**).

The secondary binding loop of textilinin-1 makes relatively few contacts with microplasmin but it does shield a small portion of the surface of microplasmin from the solvent (**Figures 4a and 4c**). The most important polar attraction is an interaction between G39O of textilinin-1 and E606OE1 of microplasmin. This suggests that the carboxylate of E606 is protonated, and/or that a water molecule(s) not visible in the electron density at 2.78 Å resolution bridges the two groups. Elevated pKa’s (as high as 8) for glutamate side-chains are rare but can occur when they are buried inside proteins [**19**], which is the case in this structure when the complex is formed. The backbone dihedral angles of G39 in both free [15] and complexed textilinin-1 are in a disallowed region of the Ramachandran plot for the other standard amino acids indicating that glycine may be the only residue capable of optimally making this bond. The side-chain of I36 of textilinin-1 is exposed to the solvent but does form hydrophobic attractions with E687, Q738 and to a lesser extent H603 of microplasmin. If the inhibitor has evolved to specifically target plasmin it is curious that a polar or charged side-chain is not in this location to optimize binding, but such a side-chain may pull R19 (P2') towards the textilinin-1 surface rather than making interactions with microplasmin, thereby weakening the overall affinity for plasmin.

The only difference in amino acid sequence in the secondary loops of textilinin-1 and aprotinin is at position 36, which is an isoleucine in textilinin-1 and a valine in aprotinin (**Figure 5**). The contact between the CD1 atom (the only difference in structure between valine and isoleucine) and plasmin is minimal with a closest approach of 3.2 Å to E687 in one complex in the asymmetric unit and 4.4 Å in the other complex in the asymmetric unit. It is therefore unlikely that this difference would even partially account for the fact that aprotinin binds 44-fold more tightly than textilinin-1 to plasmin.

**Figure 7:**
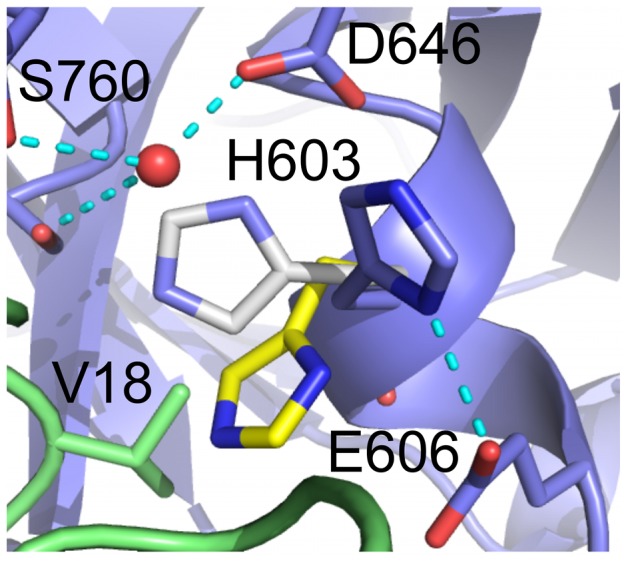
The catalytic triad of microplasmin and interactions with textilinin-1. Microplasmin, as it appears in the textilinin-1 complex, is identified by the blue carbon atoms and textilinin-1 is coloured green. The locations of the side-chain of H603 of microplasmin when bound to streptokinase (carbon atoms in white) (pdb code 1BML) and in the microplasminogen structure (carbon atoms in yellow) are overlayed. If the side-chain of H603 is located in the former orientation it would be within 1 Å of the side-chain of V18 and therefore a steric clash would exist.

In the active form of serine proteases, the Ser-His-Asp catalytic triad (S741, H603 and D646 in human plasmin) forms a compact unit with the histidine side-chain centrally located and hydrogen bonded to both the serine and aspartate. However, in both complexes of the asymmetric unit in the microplasmin-textilinin-1 crystal structure the side-chain of H603 is positioned in an alternate conformation when compared to the active conformation of the catalytic triad in serine proteases (**Figure 7**). The space that is normally occupied by the histidine side-chain is filled by a water molecule, which forms hydrogen bonds to S741OG, D646OD1 and S760O. Based on X-ray crystallography, it is not possible to unambiguously assign the position of the ND1 and NE2 atoms of the imidazolium ring of H603 (**Figure 7**). However, if the ND1 atom is oriented toward E606OD1 a hydrogen bond would be present. In either conformation, the H603 side-chain interacts through van der Waals contact with the C16-C40 bridge of textilinin-1. The side-chain of H603 has an orientation not observed in any of the other microplasmin structures previously reported. When bound to streptokinase this side-chain is in the serine protease “active” conformation [12]; **Figure 7**. However, in the microplasminogen structure this side-chain is partially rotated in a position intermediate between the active conformation and the conformation when textilinin-1 is bound [20]; **Figure 7**. The movement of this side-chain is largely attributed to a rotation of the χ_1_ dihedral angle which differs by 67° in the microplasmin-streptokinase complex and the microplasminogen structure and by -157° (*i.e.* in the opposite rotation direction compared to microplasminogen) in the microplasmin-streptokinase complex and the microplasmin-textilinin-1 complex. Modelling studies show that if the histidine side-chain is placed in its catalytically active position in the triad a steric clash would occur with the side-chain of the P1′ residue, V18, from textilinin-1 (**Figure 7**). As a result of the atypical location of the histidine side-chain there is a lack of complementarity at this site and this may partly explain why textilinin-1 binds more weakly to plasmin than does aprotinin.

### Crystal Packing in the Microplasmin-textilinin-1 Complex

In order to assess the effect of crystal packing on the overall structure of the complex a detailed analysis of the non-covalent interactions between non crystallographic and symmetry related partners is required. The buried surface area between complex A and complex B in the asymmetric unit of the mcroplasmin-textilinin-1 complex is 221 Å^2^. In this interface the side-chain of the P2' residue (R19) of textilinin-1 from complex B, which is situated in the partly open S2' subsite of microplasmin, extends out to form an ionic contact with the side-chain of D28 of textilinin-1 from complex A (3.03 Å). However, the conformation and location of the side-chain of R19 of textilinin-1 is similar in both complexes. Thus, it is unlikely that this interaction would perturb the positioning of textilinin-1 relative to microplasmin in this complex.

There are also some crystal contacts between textilinin-1 molecules and symmetry neighbours. For molecule A, there are three van der Waals contacts (3.4–3.6 Å) between the side-chains of E51 and E52 and side-chains of symmetry neighbours S51 and F47, respectively. Again it would seem these relatively few interactions would not significantly influence the docking angle of textilinin-1, especially given that the side-chain dihedral angles could easily move to prevent unfavourable interactions. For molecule B, seven side-chains form contacts with symmetry neighbours: L9 forms a van der Waals contact with P609; the side-chain of P10 forms a van der Waals contact with N769; the side-chain of D28 forms ionic bonds with the side-chains of R767 and K770 and van der Waals contacts with Pro768 and; the side-chains of E51, D52, E54, S55 and S56 form hydrogen bonds with the side-chain of S612. Again all of these are side-chain contacts but they are numerous leading us to conclude that we cannot completely rule out that crystal packing could affect the docking angle. Nonetheless, as suggested earlier, the structures of the two complexes show that the interaction between microplasmin and textilinin-1 is flexible and there is freedom of movement around the P1’ site if outside forces such as those due to crystal packing are present.

### Crystal Structure of the Trypsin-textilinin-1 Complex

**Figure 8:**
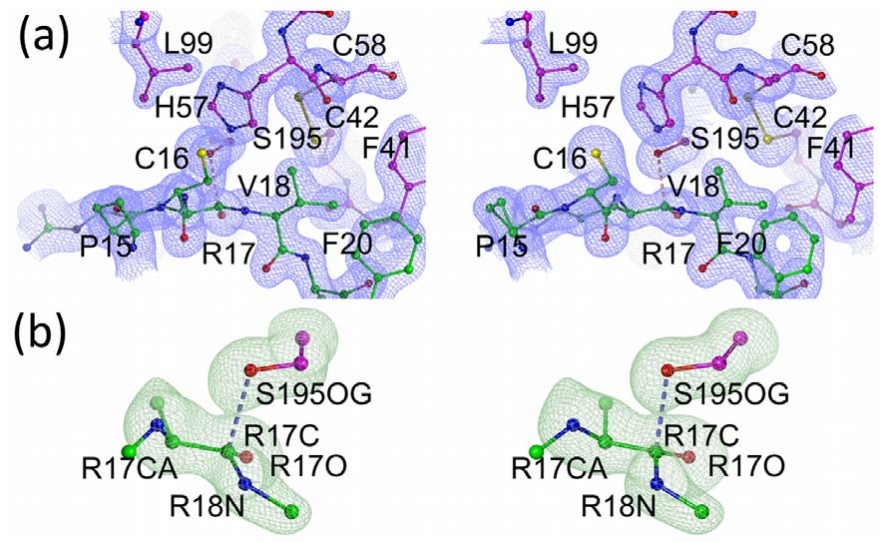
Electron density maps for the trypsin-textilinin-1 complex. (a) Stereo representation of the 2F_o_-F_c_ electron density (contoured at 1.5 σ) for the interface between textilinin-1 and trypsin. (b) omit F_o_-F_c_ electron density map after simulated annealing contoured at 3σ for R17 (textilinin-1) and S195 (trypsin) Textilinin-1 and trypsin are identified by purple and green carbon atoms, respectively. Bond angles centered around the carbonyl carbon for the tetrahedral intermediates refined to values in the range 93.0° to 119.1° with an average value of 108.6° for this complex.

**Figure 9:**
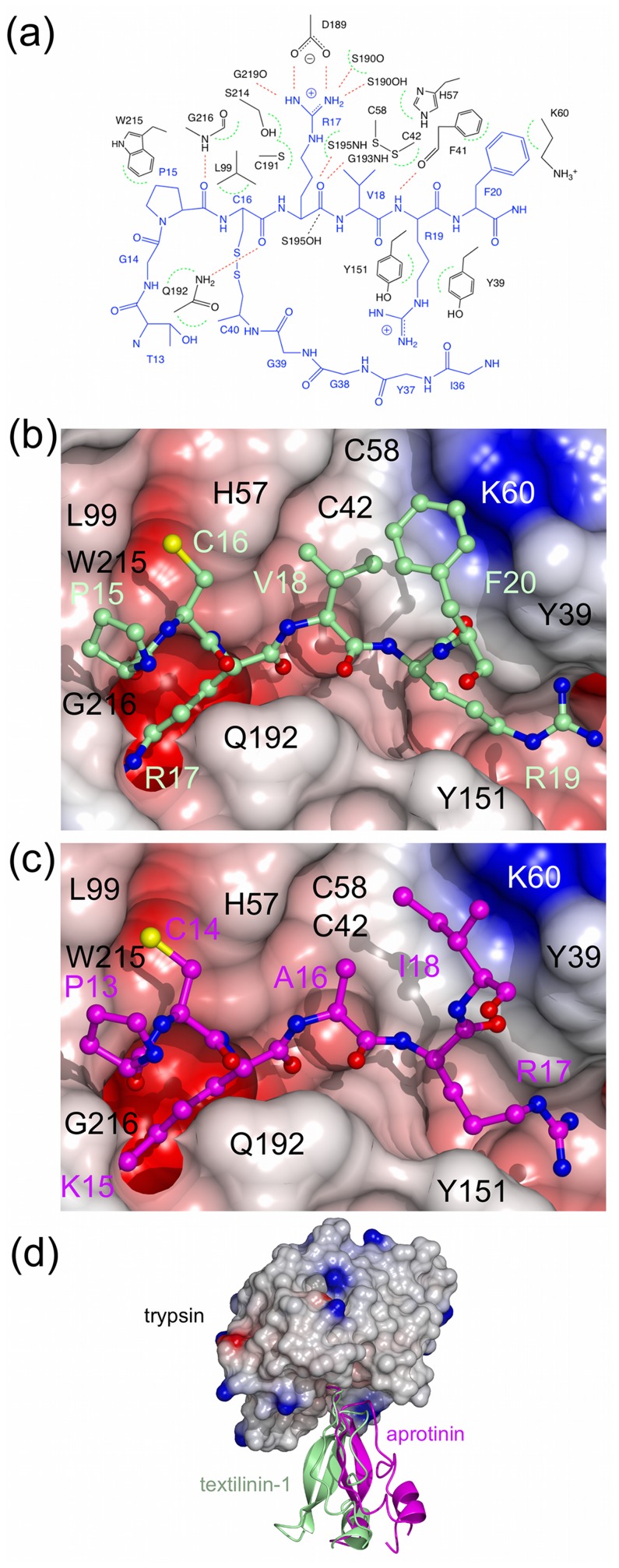
Structural images for the trypsin-textilinin-1 complex. (a) Interactions between textilinin-1 (blue) and trypsin (black). Hydrogen bonds are shown as red dashed lines. Dashed green semi-circles represent van der Waals attractions. A closer than van der Waals approach of the hydroxyl nucleophile of S195 towards the carbonyl carbon of R17 is depicted by a black dashed line. (b) Connolly and electrostatic surface of trypsin and interactions with textilinin-1 (stick model with green carbon atoms). (c) Connolly and electrostatic surface of trypsin and interactions with aprotinin (stick model with magenta carbon atoms). (d) Superimposition of the trypsin-textilinin-1 and trypsin-aprotinin complexes. Trypsin is shown as a Connolly and electrostatic surface. Textilinin-1 is in green and aprotinin in magenta.

The crystal structure of the complex between bovine trypsin and textilinin-1 has been determined to 1.64 Å resolution (**Table 2**). The 2F_o_-F_c_ electron density map for the interface between trypsin and textilinin-1 and the omit simulated annealed F_o_-F_c_ electron density for the catalytic serine of trypsin and for the P1-P1′ (R17-V18) backbone atoms for textilinin-1 are shown in **Figure 8**. The interactions between trypsin and textilinin-1 are presented in **Figures 9a and b**. The S1 specificity pocket in trypsin is filled by R17 of textilinin-1 with the side-chain forming a salt bridge with the side-chain of D189 and hydrogen bonds to S190O, S190OG and G219O. Two water molecules also fill this pocket. As in the microplasmin-textilinin-1 complex, the carbonyl oxygen of R17 extends towards the oxyanion hole and forms hydrogen bonds to two backbone amides, G193N and S195N. S195OG is 2.6 Å from the P1 carbonyl carbon of R17 of textilinin-1 (**Figure 8** **)**. Thus, as occurs in the microplasmin complex with textilinin-1 and when aprotinin is in complex with trypsin (**Figure 9c**) the reaction is arrested with the P1-P1' bond intact. The difference in this case is that the distance between the carbonyl carbon of the P1 residue and the hydroxyl oxygen of the serine nucleophile is much further apart (2.6 Å *cf* 1.6 Å).

The S1' subsite in trypsin is lined by the side-chains of F41, the catalytic H57, and the C42-C58 bridge. The side-chain of the P1' residue of textilinin-1, V18, fits in this site (**Figures 9a and 9b**). Unlike the microplasmin-textilinin-1 complex, the catalytic triad of trypsin is maintained in its catalytically active conformation with the histidine centrally located. The S2' site is a half open channel bordered by the side-chains of two tyrosine residues, Y39 and Y151. The side-chain of the P2' residue, R19, fills this site but is also exposed to solvent forming a hydrogen bond to a water molecule which bridges to H40O of trypsin. The P3' residue, F20, has one side of its aromatic ring resting against the side-chain of K60, while the remainder of the side-chain is exposed to solvent.

Superposition of the trypsin-aprotinin and trypsin-textilinin-1 complexes shows that the docking angle of the two inhibitors into trypsin differs by ∼25° (**Figure 9d**). This appears to be due to the fact that if textilinin-1 was to bind in the same orientation as aprotinin steric clashes would occur between H60 of trypsin and the P1' (V18) and the P3' (F20) and K63. Thus, the P3-P1 residues of both aprotinin and textilinin-1 slot into their sites into trypsin in an almost identical way, but the P1'-P3' residues of textilnin-1 adapt as best they can to fit into their respective sites. This is achieved in two ways: modification of backbone dihedral angles and change of docking angle of the inhibitor. Overall, the surface area that is buried in the trypsin-textilinin-1 complex is 77 Å^2^ less than in the trypsin-aprotinin complex. The overall impression is that the textilinin-1 molecule does not fit neatly into the subsites on the P1' side of the active site due to steric clashes. Nonetheless, binding is maintained due to the formation of the tetrahedral intermediate. These observations are in good agreement with the determined K_i_ values for textilinin-1 (0.42 nM) and aprotinin (6.0×10^−5^ nM) binding to trypsin [9].

### Comparison of the Structure of Free Textilinin-1 with Textilinin-1 in Complex with Trypsin and Microplasmin

**Figure 10:**
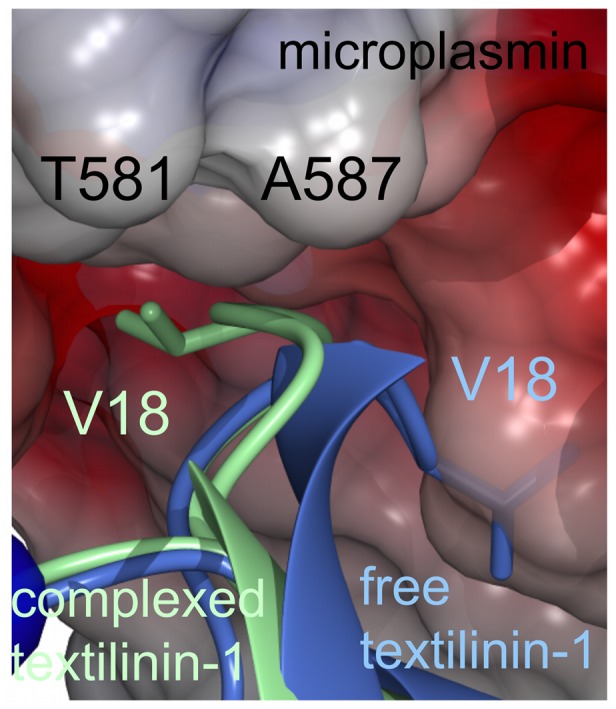
Superimposition of the crystal structure of a molecule of free textilinin-1 and the microplasmin-textilinin-1 complex. Microplasmin is shown as a Connolly and electrostatic surface. Free textilinin-1 is in blue and textilinin-1 as it exists in the microplasmin complex is in green.

The crystal structure of free textilinin-1 [15] has three protein molecules present in the asymmetric unit. Two of these molecules closely resemble the structure of textilinin-1 when bound to microplasmin or trypsin. However, the third molecule differs in that the canonical loop adopts a structure where the loop is inverted such that the side-chain of V18 is tucked into a crevice on the surface of the protein (**Figure 10**). Thus, textilinin-1 appears to possess inherent flexibility in the canonical loop of textilinin-1 that has not been observed for other aprotinin-like protease inhibitors.

## Discussion

There is a >4000 fold difference in K_i_ for textilinin-1 inhibiting plasma kallikrein compared to plasmin. The on rate is 100-fold slower and the off rate 100-fold faster than observed when textilinin-1 binds to plasmin. In plasma kallikrein the space behind the catalytic histidine is occupied by the side-chain of a tyrosine residue from the 99-loop [21], [22] effectively locking the histidine side-chain in place (**Figures 6** **and** **11**). Thus, in plasmin there is space in the S1′ site for valine, but only after the histidine has moved. However, in plasma kallikrein it does not appear to be possible for this movement to occur and it is therefore unlikely that textilinin-1 can get close enough to the active site serine (and catalytic triad) to form a tightly bound and stable tetrahedral intermediate, thereby providing an explanation for the >4000 fold difference in K_i_ value.

**Figure 11:**
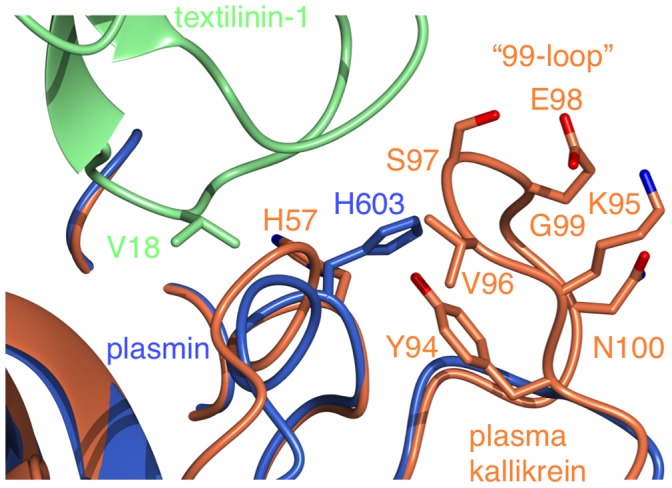
Superimposition of the catalytic subunits of plasma kallikrein and microplasmin from the microplasmin-textilinin-1 complex. The 99-loop makes a close approach to H603 and to the secondary binding loop of textilinin-1. The side-chains of Y94 and S97 and the backbone atoms of E98 and G99 of plasma kallikrein make steric clashes with H603 in this conformation. Plasma kallikrein is in brown and microplasmin (blue) from the microplasmin-textilinin-1 is in blue.

The K_i_ value for textilinin-1 binding to plasmin is 44-times higher than aprotinin. Based on the crystal structure of the microplasmin-textilinin-1 complex, a likely explanation is that, at the P1′ site, the alanine side-chain of aprotinin can fit neatly in the S1′ site of the enzyme and that the histidine side-chain is in position for catalysis. If this is the case then the interface between the enzyme and inhibitor should be sealed via hydrophobic interactions. On the other hand, the movement of the histidine side-chain in the complex with textilinin-1 reduces the surface complementarity at this site. Furthermore, the introduction of a water molecule in the location of the histidine side-chain creates a polar interface, which would not be a favoured interaction with the non-polar side-chain of the P1′ valine of textilinin-1.

An important property in terms of its therapeutic potential is that textilinin-1 has a much faster off-rate for plasmin as compared to aprotinin (**Table 1**). There is as yet no crystal structure for aprotinin in complex with plasmin, but the P1' residue in aprotinin is alanine, which is smaller by two methyl groups than V18 in textilinin-1. Therefore, it is likely that when aprotinin binds to plasmin the catalytic triad remains intact. In the case of textilinin-1 bound plasmin, dissociation of the complex could be facilitated by the movement of the histidine back into its position in the catalytic triad, rapidly releasing the inhibitor from plasmin. A feature of textilinin-1 is that the canonical loop in the free molecule can adopt an inverted structure that is stabilized by interactions between V18, T12 and I35 [15]. This flexibility may be a property of textilinin-1 that can facilitate its rapid release from the enzyme. Overall the explanation of tighter specificity and faster off rate that textilinin-1 exhibits compared to aprotinin appears to lie in the identity of the P1' residue, which is the larger valine in textilinin-1 (*cf* alanine in aprotinin). Another factor that may contribute to an enhanced binding specificity textilinin-1 has is the presence of the bulky phenylalanine side-chain at the P3' site (*cf* isoleucine in aprotinin). This may preclude textilinin-1 from optimally binding to serine proteases that have a protuberance at this site, as is observed in the trypsin structure.

Adverse effects due to the immunogenicity of textilinin-1 and other Kunitz-type inhibitors from snake venoms are considered a problem with the use of such molecules as therapeutic agents. In the case of aprotinin, effects can range from mild skin rashes to, in rare cases, anaphylaxis [23]. However, the most severe allergic reactions (<0.75% of cases) are generally restricted to those occurring upon re-exposure to aprotinin [24]. One approach to overcoming this problem is to synthesize mutant inhibitors with the aim of removing potential immunogenic epitope(s) (as has been suggested for aprotinin [25]) but maintaining the critical residues that are responsible for selective binding to the target. Another approach to drug design may be to graft the primary binding loop of textilinin-1 onto a smaller molecule which would be non-immunogenic.

### Conclusions

The more rapidly reversible inhibition of plasmin by textilinin-1 in comparison with aprotinin (**Table 1**) would be expected to result in a faster recovery of plasmin activity after cessation of treatment, leading to a decreased tendency to develop post-operative thrombosis. Compromising fibrinolysis for longer than necessary to stem blood loss would be expected to result in adverse thrombotic effects. It is well established that active fibrinolysis is necessary to prevent such adverse effects [26], [27]. Other side-effects of aprotinin may be due to its ability to inhibit a range of serine proteases involved in blood clotting and other physiological processes. The more reversible binding and better specificity of textilinin-1 compared to aprotinin suggest that this molecule may have improved pharmaceutical properties over aprotinin.

The sequence of textilinin-1 is novel. An extensive BLAST search of all Kunitz-type protease inhibitor sequences failed to find a match with the RVRF motif in the P1-P3' sites found in textilinin-1 (except for textilinin-2, also found in the Australian brown snake). The observation that the histidine side-chain is out of its position in the catalytic triad in the microplasmin complex and the flexible nature of the canonical loop in textilinin-1 could not have easily been predicted by molecular modelling. The atypical positioning of the histidine side-chain is not an unprecedented observation as it has been previously seen in the crystal structure of complement protease factor D [28]. In this enzyme the movement of this side-chain (from inactive to active state) can only be induced by C3b-bound factor B and it is suggested that this movement is the reason for its high specificity for factor B as a substrate. That plasmin can also adopt this unusual configuration of the catalytic triad in the presence of an experimental drug (textilinin-1) suggests that rational approaches could be employed to design or synthesize compounds that have greater selectivity and potency and possess highly favourable pharmacokinetic properties.

## Materials and Methods

### Protein Concentration Determination

The concentration of the reconstituted plasmin was determined by active site titration with *p*NPGB as described by Chase and Shaw [29]. Bovine lung aprotinin was purchased as Trasylol® from Bayer Corporation (Germany). The stated concentration of 1.4 mg/mL (215 µmol/L; 10,000 KIU/mL) was confirmed by active site titration with plasmin and *p*NPGB [29], [30], assuming 1∶1 stoichiometry. The molar concentration of aprotinin was also determined using the absorbance of the solution at 280 nm and the E^1%^_280_ calculated from the amino acid composition [31], and found to be in excellent agreement with the active site titration. Textilinin-1 was cloned from *Pseudonaja textilis* venom gland RNA by RT-PCR [32], and expressed and purified under contract by Hospira Ltd, Australia for QRxPharma Pty Ltd. The molar concentration of the stock textilinin-1 solution (212 µmol/L) was determined by active site titration and UV spectroscopy as described above for aprotinin.

### Reagents, Enzymes and Inhibitors

Chromogenic substrates, S-2251 for plasmin, S-2765 for trypsin, S-2302 for plasma kallikrein were purchased from Chromogenix, Sweden. Urokinase was from Dade Behring (Germany). All other reagents and enzymes were purchased from Sigma-Aldrich MO, USA. Human plasmin was a gift from Dr. J. C. Lormeau (Sanofi, France).

### Enzyme Inhibition Assays and Kinetic Data Analysis

Inhibition data were obtained by monitoring *p*-nitroaniline formation in reactions initiated by adding a 10 µL aliquot of enzyme to varying inhibitor concentrations in a 1 mL reaction mixture containing the appropriate chromogenic substrate, 0.1 M Tris-HCl, pH 7.4, 0.01% Tween-80 at 25°C.

The resulting progress curves (**Figure 1**) were analyzed by simultaneous regression using the numerical integration software DynaFit (BioKin Ltd http://www.biokin.com/dynafit/). This program allows the statistical discrimination between alternative models and we compared a simple one-step model (Scheme 1) with the two-step model of Morrison and Walsh (Scheme 2, [33]).
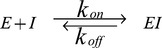
(1)

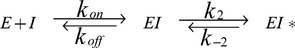
(2)


In each case the simpler one-step model was preferred based on the Akaike weights [34] and the variance ratio (F) test. The parameter estimates (*k_on_* and *k_off_*) and their associated errors are shown in **Table 1**. The error estimates of the dissociation constants (*K_i_* = *k_off_*/*k_on_*) were calculated from the square root of the sum of the squares of the cvs of the rate constants. In the case of aprotinin inhibition of trypsin the estimate of the dissociation rate constant (*k_off_* ∼ 10^−9^ s^−1^) was associated with a very large error indicating that it was not well determined. When the data were re-analysed comparing the one step model with an alternative model of irreversible inhibition (*k_off_* = 0), the latter was statistically selected with the *k_on_* value shown in **Table 1**.

### Expression, Purification and Crystallization of the Complexes

A plasmid containing human microplasminogen was transformed into *Escherichia coli* BL-21(DE3) competent cells where the protein was expressed in inclusion bodies. The protein was solubilized and refolded according to a method described previously [20]. The microplasminogen was purified by Sephacryl S-300 size exclusion chromatography. Peaks were assayed using the chromogenic substrate H-D-valyl-L-leucyl-L-lysine-p-nitroaniline (S-2251). The peak containing correctly folded (0.3 mg/ml) microplasminogen was incubated with five fold excess of textilinin-1 and 25% glycerol before mixing with urokinase immobilized on Sepharose beads. These were prepared by taking 1 g of freeze-dried CNBr-activated Sepharose 4B beads that were swollen for 15 min in 1 mM HCl and then washed on a sintered glass filter with the same solution. The final volume of the mixture after washing was 200 mL. 0.5 mL of 1000 U/mL urokinase-type plasminogen activator concentrated to 2.7 mg/mL (as determined by the BCA assay) was pipetted into 9.5 mL of 0.1 M NaHCO_3_ (Sigma-Aldrich) pH 8.3 and 0.5 M NaCl. This solution was then mixed with the swollen beads and centrifuged at 3000 rpm for 15 min. The supernatant was then decanted. 10 mL of blocking agent consisting of 0.2 M glycine pH 8.0 was mixed for four hours with the gel to block the remaining active groups, and then centrifuged at 3000 rpm for 15 min and the supernatant decanted off. The gel was next washed with 50 mM Tris-HCl pH 7.4 and again centrifuged at the same speed for 10 min. The complex was separated from the beads by centrifugation (3000 rpm for 15 min). The supernatant was inactive, but showed the two proteins on SDS-PAGE. Prior to crystallization this complex was concentrated to 6 mg/ml.

Bovine trypsin (Product No. T1426, Sigma-Aldrich) was purchased as a salt-free lyophilized powder. To form the trypsin-textilinin-1 complex lyophilized trypsin (0.5 mg/ml) was dissolved in a solution containing textilinin-1 in 25 mM Tris–HCl pH 7.8, in the molar ratio 1∶1.1 (enzyme:inhibitor). The complex was purified by loading onto a HiLoad Superdex 200 column (Prep Grade) equilibrated with 25 mM Tris–HCl pH 7.4 and 50 mM NaCl. This sample showed 95% inhibition after purification.

Crystals of the microplasmin-textilinin-1 complex were obtained by the hanging drop vapour phase diffusion method at 17°C within two weeks of setting up the experiment. The well solution consisted of 0.17 M ammonium sulfate, 25.5% PEG 4000 and 15% glycerol (no buffer was in this solution). The drop comprised 1 µl of well solution and 1 µl of complex. A crystal was directly transferred to a cryostream (100 K) without addition of cryoprotectant. Crystallization of the trypsin-textilinin-1 complex was also by the hanging drop method but the experiment was undertaken at 4°C and using a protein concentration of 10 mg/mL. Crystals took up to one month to appear in the drops. The well solution consisted of 0.1 M HEPES-Na pH 7.5 and 1.4 M tri-sodium citrate dihydrate. The drop comprised 1 µl of well solution and 1 µl of complex. Crystals were transferred from their growth drop to a new drop containing 70% (v/v) well solution and 30% (v/v) glycerol and soaked for 5 min in this solution before being transferred to the cryostream (100 K).

### Data Collection and Structure Determination

Data sets for both complexes were collected using a Molecular Structure Incorporated FR-E X-ray generator operated at 45 kV and 45 mA and an R-Axis IV++ imaging plate detector. For the microplasmin-textilinin-1 complex, 200 oscillation images with an oscillation range of 0.5° were collected. The exposure time for each image was 180 s. For the trypsin-textilinin-1 complex, 150 oscillation images with an oscillation range of 0.5° were collected. The exposure time for each image was 360 s. Data for the microplasmin-textilinin-1 complex were integrated, scaled and merged using Crystalclear 1.3.6 [35]. Data for the trypsin-textilinin-1 complex were integrated, scaled and merged using HKL2000 [36]. The structures of both complexes were solved by molecular replacement using EPMR [37]. The coordinates of microplasmin from the complex with streptokinase (pdb code 1BML) and bovine trypsin (pdbcode 3PTN) were used as starting models for the respective complexes. The initial electron density maps revealed textilinin-1 bound in the active site of each the proteases. Textilinin-1 was subsequently docked into this space. Refinement of both complexes was carried out initially using REFMAC5 [38] followed by Phenix [39], and model building and structural analysis were performed with Coot [40] with MOLPROBITY [41], respectively. Accessible surface areas were calculated using the method of Lee and Richards [42]. To determine the docking angles of textilinin-1 to microplasmin the microplasmin structures were superimposed on each other and the coordinates of the textilinin-1 rotated until they superimposed.

### Accession Codes

The atomic coordinates and structure factors have been deposited into the Protein Data Bank with accession codes 3UIR and 3D65.
